# Uterovaginal prolapse in a primigravida presenting in active first stage of labor: a case report

**DOI:** 10.1186/s13256-022-03358-y

**Published:** 2022-04-08

**Authors:** Bezza Kedida Dabi, Demisew Amenu Sori, Fanta Asefa Disasa

**Affiliations:** Department of Obstetrics and Gynecology, Jimma Medical Center, Jimma University, Jimma, Ethiopia

**Keywords:** Uterovaginal prolapse, Third-trimester pregnancy, Primigravida, Active first stage of labor, Case report, Ethiopia

## Abstract

**Background:**

Uterovaginal prolapse is the descent of the uterus and vagina down the birth canal toward the introitus. The occurrence of uterovaginal prolapse in a primigravida is very rare. It can cause preterm labor, fetal demise, spontaneous abortion, postpartum hemorrhage, maternal urinary complications, sepsis, and death. This case report presents the rare occurrence of uterovaginal prolapse in a primigravida woman with no major risk factors identified for prolapse, who presented in active first stage of labor and delivered vaginally.

**Case presentation:**

A 30-year-old Oromo primigravida woman who did not remember her last normal menstrual period but claimed amenorrhea of 9 months duration presented with the urge to bear down of 12 hours duration and passage of liquor of 8 hours duration. She was referred from the local health center to Jimma Medical Center with a diagnosis of prolonged labor. At presentation, she was in active first stage of labor with cervix 5–6 cm and fetal heartbeat was negative. She was followed for the progress of labor, and 4 hours after admission to the labor ward, she delivered a freshly dead male neonate weighing 3000 g. Her postpartum period was uneventful, and she was discharged on her third postpartum day and referred after 6 weeks to the outpatient department.

**Conclusion:**

Uterovaginal prolapse occurring in primigravida and during labor at first recognition is very rare, with congenital weakness being a possible underlying pathology. Management of uterovaginal prolapse during labor should be individualized on the basis of fetal condition and the severity of prolapse. For a patient with pelvic organ prolapse in labor, expectant management is a good option when there is no severe edema resulting in obstructed labor, as in our case, where the patient delivered vaginally and the prolapse resolved postpartum.

## Background

Uterine prolapse during pregnancy is a rare condition, with an incidence of 1 per 10,000 to 1 per 15,000 deliveries [1, 2]. It can result in preterm labor, spontaneous abortion, fetal demise, maternal urinary complications, maternal sepsis, and death [1]. Most of the reported patients were managed conservatively during pregnancy by reducing the prolapse followed by rest in the Trendelenburg position [3]. The rate of adverse pregnancy outcomes has decreased dramatically since the past century, probably due to changes in obstetric practice and advances in neonatology. The overall fetal mortality rate in women with pelvic organ prolapse (POP) in pregnancy was 22% in 1941 [4]. However, eight perinatal deaths have been reported since 1990, all from developing countries [4]. We report a case of uterovaginal prolapse in a primigravida woman, in her third trimester of pregnancy, presenting in active first stage of labor.

## Case presentation

A 30-year-old Oromo primigravida woman who did not remember her last normal menstrual period, but claimed amenorrhea of 9 months, presented to Jimma Medical Center with an urge to bear down of 12 hours duration and passage of liquor of 8 hours duration. She received antenatal care at a local health center two times and was referred from there with a diagnosis of prolonged labor. She also complained of decreased fetal movement of 2 days duration and prolapsed mass per vagina while she was in an ambulance on the way to the Jimma Medical Center. She had a history of small prolapsed mass per vagina before pregnancy when she was walking, which reduced when she lay down, but this did not worry her and it disappeared during pregnancy. Her personal, familial, and medical histories were unremarkable. The pregnancy was unplanned but wanted and supported.

**Fig. 1:**
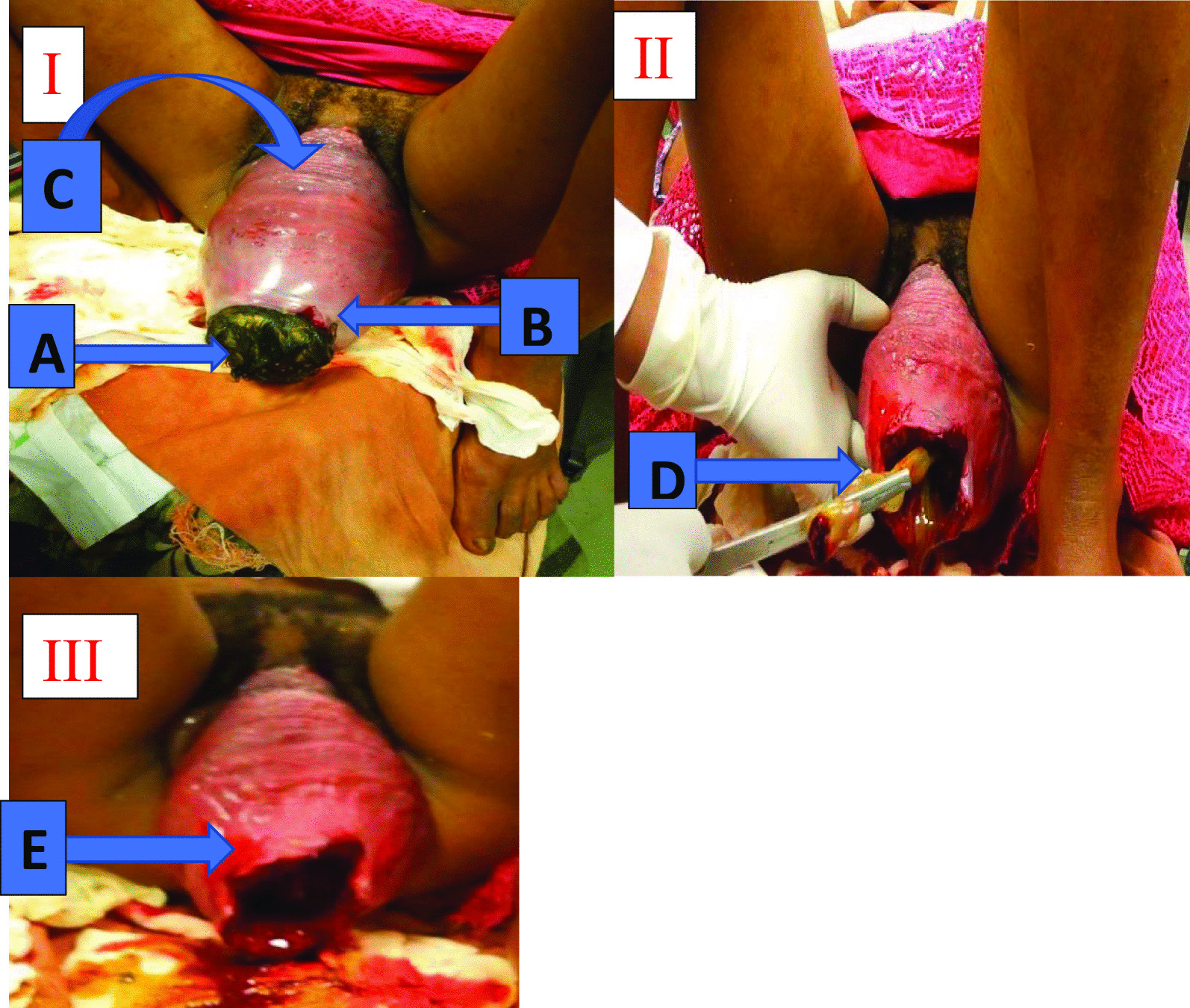
Uterovaginal prolapse. **A** Fetal head in the prolapsed uterus. **B** Cervix tightened around the fetal head with laceration at 2 o’clock. **C** Prolapsed anterior vaginal wall. **D** Cord with placenta inside the prolapsed uterus. **E** Prolapsed uterus after delivery of the placenta and the fetus

Upon arrival, she was in labor pain, and her vital signs were blood pressure 120/80 mmHg, pulse rate 90 beats per minute, respiratory rate 22 breaths per minute, and body temperature 36.5 °C. Pertinent findings were on the abdomen: 26-week-sized gravid uterus, fundus occupied by soft bulky mass that was breech. The lie was longitudinal, and the presentation was cephalic. Fetal heart sounds were absent on auscultation with Pinard fetoscope and confirmed by ultrasound. She had three contractions in 10 minutes lasting for 40–60 seconds. Ultrasound revealed a singleton intrauterine pregnancy; the fetus was 38 weeks, the placenta was fundal anterior, and no gross congenital anomaly was seen. Pelvic examination showed a prolapsed vaginal wall with its rugae visible on the anterior vaginal wall. Edematous, irreducible cervicouterine prolapse and fetal head protruding through prolapsed cervix were visible outside the vagina. Cervix was 5–6 cm dilated, edematous, and lacerated at 2 and 10 o’clock position, but it was not bleeding (Fig. 1). There was grade 3 meconium-stained amniotic fluid.

Owing to the combination of third-trimester pregnancy, intrauterine fetal death, active first stage of labor, and uterovaginal prolapse, she was tested for blood group and Rh (A^+^) and hematocrit (44%), and then she was followed for the progress of labor. She entered the second stage 3 hours after admission to the labor ward, was encouraged to push, and delivered a freshly dead male neonate weighing 3000 g after 1 hour in the second stage of labor. There was a cervical tear at 10, 2 o’clock, but there was no significant bleeding and it was stopped by compression with a pack. Upon examination, no gross fetal anomaly was seen, placental weight was 600 g, and cord length was 50 cm with two arteries and one vein. After delivery of the placenta, the patient was put in the Trendelenburg position, prolapse was elevated, and ice packs were applied to decrease edema. The size of the prolapse gradually reduced, the edema subsided, and manual reduction of the prolapse was performed on the first postpartum day. The uterus was involuted; cervix was at the level of introitus. She was discharged on the third postpartum day and scheduled for follow-up at 6 weeks. The patient was contacted on phone at 6 weeks and 3 months postpartum, but she could not come back to hospital for personal reasons; she claimed she has no prolapsed mass through the vagina.

## Discussion

This case report presents a rare case of uterovaginal prolapse in primigravida presenting as an emergency in active first stage of labor after amenorrhea of 9 months duration. Uterovaginal prolapse is common in women who are multiparous and of older age; however, it rarely occurs during pregnancy and in primigravida women [5, 6]. The cause of uterine prolapse during pregnancy may be multifactorial, including multiparty, age, malnutrition, race, vaginal delivery, short interval between consecutive pregnancies, physiologic change of pregnancy causing cervical elongations, and previous history of prolapse [1, 2, 7–12]. Pelvic organ prolapse (POP) presenting before pregnancy is less common and resolves during pregnancy, but the acute onset of POP in pregnancy is more common [7]. Acute onset of POP during pregnancy is often first recognized during third-trimester pregnancy [7, 12].

Our patient had a history of small prolapsed mass through the vagina before pregnancy, which disappeared during pregnancy and acutely appeared during labor. Our patient probably had asymptomatic preexisting prolapse that was aggravated by the pregnancy and course of labor. Increased cortisol and progesterone during pregnancy and increased intraabdominal pressure with labor may have contributed to uterovaginal prolapse. Acute onset of POP most frequently occurs in the second trimester of pregnancy. However, it was first recognized in labor in some case reports [4], similar to our patient’s presentation. Prolapse that exists before pregnancy usually resolves by the end of second trimester [11], which is similar to our patient’s presentation. POP in primigravida (Table 1) is a rare event [1, 5, 6, 8]. A small degree of prolapse is normal in nulliparous women, and the degree of prolapse increases with parturition [13], as it occurred in our patient. A small case–control study comparing nulliparous with primigravida showed that pregnant women have more vaginal prolapse [13]; another study reported younger women with genital prolapse having lower collagen concentration than age-matched controls [14]. Our patient is young, and it is possible that she has lower collagen concentration, although we did not take a tissue biopsy to assess collagen concentration. The fact that uterine prolapse does occur in primigravida without preexisting descent seems to suggest that congenital weakness in pelvic support structure could be an underlying pathology.

**Table 1:** Review of literature on pelvic organ prolapse in pregnancy

Study	Year	Age	Parity	Mode of delivery	Birth weight (g)	Complications reported	Follow-up
Ghose *et al*. [8]	2012	26	Primigravida	Spont.Del.*	2100	None reported	NA
Zeng *et al*. [7]	2018	27	G3P2	Cesarean section	2480	None reported	NA
Zeng *et al*. [7]	2018	33	G2P1	Spont.Del.*	2680	None reported	POP recurred
Cingillioglu *et al*. [10]	2010	29	G3P2	Cesarean section	2960	None reported	POP resolved
Meydanli *et al*. [9]	2006	30	G6P5	Cesarean section	2300	Cesarean hysterectomy, cervical dystocia	No vaginal vault prolapse
Mohamed-Suphan and Ng [3]	2012	26	G4P2	Cesarean section	3100	None reported	POP persisted
Kim *et al*. [1]	2016	32	Primigravida	Spont.Del.*	2670	None reported	POP resolved
Saha *et al*. [2]	2015	28	G4P3	Expelled abortus	NA	Urine retention, abortion	POP resolved
Yousaf *et al*. [12]	2011	35	G2P1	Spont.Del.*	2400	Cervical laceration, hydronephrosis	POP persisted
Kart *et al*. [15]	2010	21	G4P3	Spont.Del.*	860	Preterm delivery	POP persisted
Kart *et al*. [15]	2010	36	G3P2	Spont.Del.^*^	3300	None reported	POP persisted
Buyukbayrak *et al*. [6]	2010	19	Primigravida	Spont.Del.^*^	3200	None reported	POP resolved
Ishida *et al*. [5]	2014	31	Primigravida	Cesarean section	3230	Cervical edema	POP resolved
Our patient	2012	30	Primigravida	Spont.Del.*	3000	Cervical laceration	NA

Spont.Del.*, spontaneous delivery; NA, not applicable

The main antepartum complication in pregnant women with prolapse is preterm labor [11]. In our patient, fundal height was 26 weeks sized, and this was due to a significantly prolapsed uterus (Fig. 1). Our patient also claimed amenorrhea of 9 months duration, fetus was 38 weeks, and the birth outcome was 3000 g freshly dead male neonate (Table 1). Fetal death and maternal morbidity are rarely reported complications [11]. A systematic review reported only four fetal deaths, and all of them were from developing countries [4]. Even though there was fetal death in our patient, respiratory failure secondary to meconium aspiration syndrome is a possible cofactor as there was grade 3 meconium-stained liquor. Intrapartum complications of uterovaginal prolapse include the inability of cervical dilatation, cervical dystocia due to edema, cervical laceration, and obstructed labor with the possible risk of uterine rupture [3]. Among the above complications, our patient had cervical lacerations at 2 and 10 o’clock (Fig. 1).

Management of the prolapse should be individualized, and the managing obstetrician must have possible complications in mind. Bed rest in the Trendelenburg position should be advised to decrease edema and displacement of the uterus [11]. Good genital hygiene is imperative, and local antiseptics should be applied in the event of ulcerations or infected cervix [11]. Conservative management during pregnancy is the treatment of choice because the prolapse usually resolves spontaneously following delivery [1]. Conservative management includes genital hygiene and bed rest in slit Trendelenburg position [15]. POP can be successfully managed by a pessary throughout the pregnancy until the onset of labor [3, 4, 6]. Women with severe prolapse are at increased risk of cesarean section due to obstructed labor; however, vaginal delivery is not contraindicated [4]. Our patient had a successful vaginal delivery (Table 1). A primary cesarean section is an option in case of severe POP with acute onset during pregnancy as it seems to be protective for prolapse after delivery [4].

## Conclusion

Uterovaginal prolapse occurring in primigravida and during labor at first recognition is very rare, with congenital weakness being a possible underlying pathology. Management of uterovaginal prolapse during labor should be individualized on the basis of fetal condition and the severity of prolapse. For a patient with pelvic organ prolapse in labor, expectant management is a good option when there is no severe edema resulting in obstructed labor, as in our case where the patient delivered vaginally and the prolapse resolved postpartum.

## Data Availability

Data sharing does not apply to this article as no datasets were generated or analyzed during the current study.
